# Copy-number variation in *BMPR2 *is not associated with the pathogenesis of pulmonary arterial hypertension

**DOI:** 10.1186/1471-2350-10-58

**Published:** 2009-06-16

**Authors:** Jennifer A Johnson, Cindy L Vnencak-Jones, Joy D Cogan, James E Loyd, James West

**Affiliations:** 1 Department of Medicine, Vanderbilt University Medical Center, Nashville, TN, USA; 2 Department of Pediatrics, Vanderbilt University Medical Center, Nashville, TN, USA; 3 Department of Pathology, Vanderbilt University Medical Center, Nashville, TN, USA

## Abstract

**Background:**

Copy-number variations (CNVs) are structural variations in the genome involving 1 kb to 3 mb of DNA. CNV has been reported within intron 1 of the *BMPR2 *gene. We propose that CNV could affect phenotype in familial and/or sporadic pulmonary arterial hypertension (PAH) by altering gene expression.

**Methods:**

97 human DNA samples were obtained which included 24 patients with familial PAH, 18 obligate carriers (*BMPR2 *mutation positive), 20 sporadic PAH patients, and 35 controls. Two sets of primers were designed within the CNV, and two sets of control primers were designed outside the CNV. Quantitative PCR was performed to quantify genomic copies of CNV and control sequences.

**Results:**

A CNV in *BMPR2 *was present in one African American negative control subject.

**Conclusion:**

We conclude that the CNV in intron 1 in *BMPR2 *is unlikely to play a role in the pathogenesis of either familial or sporadic PAH.

**Trial Registration:**

NIH NCT00091546.

## Background

Pulmonary arterial hypertension is a progressive disease characterized by obstruction of pre-capillary pulmonary arteries. Symptoms of PAH include fatigue and shortness of breath. Sustained pulmonary arterial hypertension eventually leads to right-sided heart failure and death. In 2000, mutations in *BMPR2 *(bone morphogenic protein receptor type 2) located on chromosome 2 were shown to cause familial PAH[1]. The *BMPR2 *gene spans 190 kb but encodes only a 4 kb transcript. It contains 13 exons and a large first intron composed of over 90 kb with frequent repetitive sequences. *BMPR2 *mutations are present in over 80% of patients diagnosed with familial PAH and are responsible for some cases of idiopathic PAH. Although the discovery of the *BMPR2 *mutation has enhanced our understanding of PAH, there are several important characteristics of the disease which remain unexplained. For example, the lifetime risk of developing PAH with the *BMPR2 *mutation is less than 20%, anticipation is observed, and there are both familial and sporadic patients without the *BMPR2 *mutation who develop PAH[2]. Recently, penetrance has been linked to levels of *BMPR2*; unaffected carriers (patients with a *BMPR2 *mutation who do not have PAH) have higher levels of wild-type *BMPR2 *allele transcript than patients with *BMPR2 *mutations and PAH[3]. This finding suggests that alterations of gene expression may be relevant to PAH phenotype.

CNVs contribute to genetic variation through insertion, deletion, or inversion of DNA. Examples of diseases in which CNV participates mechanistically include DiGeorge syndrome, Angelman syndrome, Alzheimer's, autism, and schizophrenia[4]. Cystic fibrosis is an example of a pulmonary disease where CNV modulates gene expression. In transgenic mice, deletion of DNase I hypersensitive site in intron 1 did not change CFTR (cystic fibrosis transmembrane conductance regulator) expression in the lung, but it decreased expression of CFTR in the intestine by about 60%. Therefore, CNV in intron 1 in the CFTR gene acts as a regulatory element and is required for normal CFTR expression in the intestinal epithelium[5].

Recent advancements in array technology have allowed investigators to perform genome-wide assessments of genetic variation. To date, there are well over twenty thousand CNVs listed in the Database of Genomic Variants. The Database of Genomic Variants lists seven partially overlapping CNVs within intron 1 of *BMPR2 *on chromosome 2 consisting of a deletion of a several kilobase region[6]. Since intron 1 can contain regulatory elements, as demonstrated in cystic fibrosis, CNV in intron 1 of *BMPR2 *may be an important modifier in PAH. We propose that CNV in intron 1 of *BMPR2 *could affect penetrance, anticipation, or disease phenotype in familial and/or sporadic PAH.

## Methods

**Figure 1:**
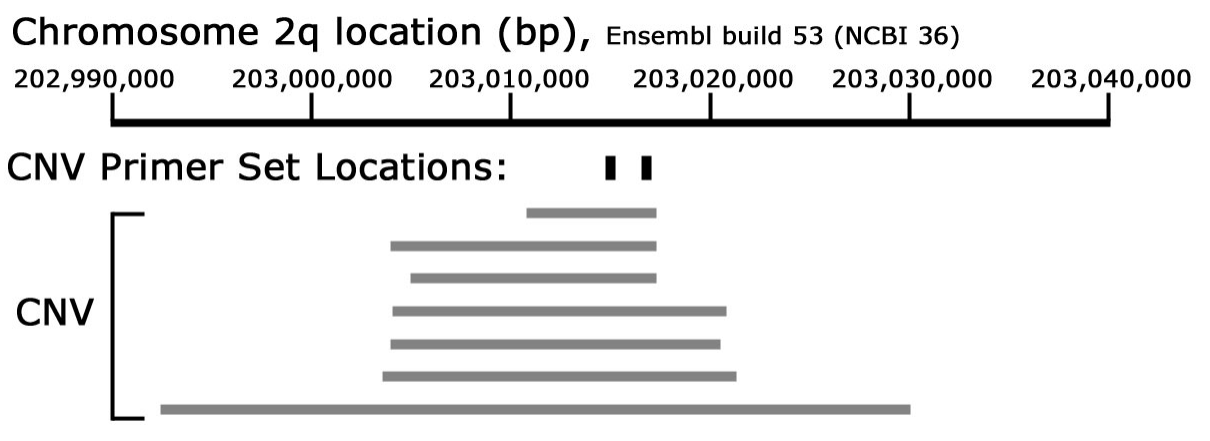
**CNV and primer set location in the *BMPR2 *genome**.

The Database of Genomic Variants was queried for CNV within the *BMPR2 *gene. The database identified seven CNVs in intron 1 at locus 2q33.1: 202,992,448 – 203,030,002. The CNVs were partially overlapping, but had different endpoints (Figure 1). They were discovered using a variety of methodologies [6-12]. All seven CNV studies used samples from the International HapMap Project which includes individuals of European, Yoruba, Chinese, and Japanese ancestry.

We designed two sets of primers within the overlapping CNV region: CNV set 1 (GCATTGAAAGTGGGATAATGG, TGAAATTTGAGGATAACAATTTTAAG) and CNV set 2 (TAATTGCCTTCAGAGCAGGG, CCAACATTTGTCAAGGATGC). We designed two sets of control primers outside the CNV (one control primer set was placed within exon 1 of *BMPR2 *and the other control primer set was placed within exon 1 of *BMPR1a*): CNV set 1 (TTGTGATTCGCTCACAGGAG, AGAGGCTGCCCCTTCTAGTC) and CNV set 2 (TGGTAAAGGCCGATATGGAG, ATGTTTTCATGGCGCATTAG) (Figure 1).

Human genomic DNA was obtained with informed consent from 97 patients with approval from the Institutional Review Board at Vanderbilt University. Genomic DNA was isolated from whole blood using the Roche MagNA Pure LC DNA purification system (Roche Molecular Biochemicals, Indianapolis, IN). Of the 97 samples, there were 24 patients with familial PAH, 18 obligate carriers (*BMPR2 *mutation positive), 20 sporadic PAH patients, and 35 controls. Obligate carriers are patients without pulmonary hypertension, but with *BMPR2 *mutations. Controls were either married-in spouses (7), unaffected family members negative for the *BMPR2 *mutation (13), or unrelated healthy individuals (14). There were 21 families with familial PAH included, representing on average 113 *BMPR2 *alleles. In efforts to evaluate penetrance and anticipation, we included seven parent-child dyads. 93 patients were Caucasian and 4 patients were African American.

DNA samples were diluted to 2 ng/ml. Quantitative PCR with SYBR Green was performed to quantify genomic copies of CNV and controls. Samples were run in triplicates on an Applied Biosystems StepOnePlus machine. A dilution series was performed to quantify primer efficiency.

## Results and Disscusion

**Figure 2:**
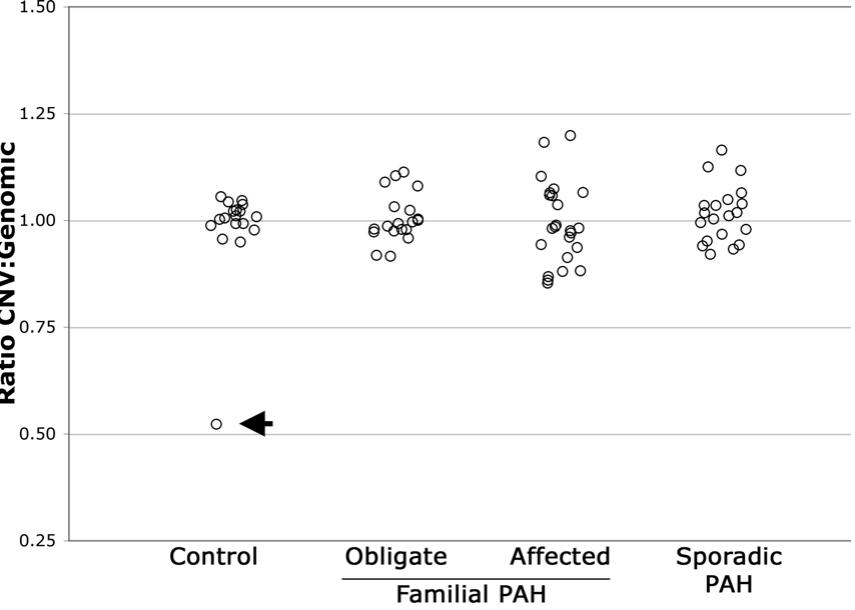
**Ratio of CNV to genomic DNA in the control, obligate, affected, and sporadic PAH patients**.

CNV in *BMPR2 *was present in one out of 97 samples. The CNV was in an African American control and consisted of a loss of one copy of the CNV region. Figure 2 represents the ratio of CNV to genomic DNA in the control, obligate, familial, and sporadic patients. Small variation from a ratio of 1.0 is caused by noise inherent to the quantitative PCR process. Since the reported CNV from the HapMap Project contained individuals of European, Yoruba, Chinese, and Japanese ancestry, it is likely that the CNV in *BMPR2 *in our patient is attributable to an ethnic basis.

## Conclusion

The recent discovery of differences in levels of *BMPR2 *allele transcript among affected PAH patients and unaffected carriers led us to look for a CNV within the *BMPR2 *gene which could affect gene expression. We succeeded in identifying CNV in *BMPR2 *in intron 1 in one patient; however, the patient was a control subject. It is unlikely that CNV within *BMPR2 *intron 1 plays a role in the pathogenesis of either familial or sporadic PAH.

## List of Abbreviations

CNV: copy-number variation; *BMPR2*: bone morphogenic protein receptor type 2; PAH: pulmonary arterial hypertension; CFTR: cystic fibrosis transmembrane conductance regulator

## Competing interests

The authors declare that they have no competing interests.

## Authors' contributions

JJ drafted the manuscript and performed quantitative PCR, CV participated in the design of the study, JC participated in the design of the study, JL participated in the design of the study, JW participated in the design of the study and performed the statistical analysis. All authors read and approved the final manuscript.

## Pre-publication history

The pre-publication history for this paper can be accessed here:


